# Is FIB-4 Index an Independent Risk Factor for Hematoma Expansion in Acute Intracerebral Hemorrhage? A Retrospective Multicenter Observational Cohort Study

**DOI:** 10.3390/jcm15124512

**Published:** 2026-06-11

**Authors:** Buket Tugan Yıldız, Mine Hayriye Sorgun, Dicle Seray Muratoğlu, Elif İpek Gencer Mutlu, Mustafa Gökçe, Canan Togay Işıkay

**Affiliations:** 1 Department of Neurology, School of Medicine, Kahramanmaras Sutcu Imam University, Kahramanmaras 46000, Turkey; 2 Department of Neurology, School of Medicine, Ankara University, Ankara 06100, Turkey

**Keywords:** FIB-4 index, hematoma expansion, liver fibrosis, intracerebral hemorrhage

## Abstract

**Background/Objectives:** The FIB-4 index is a laboratory test for predicting liver fibrosis. The aim of this study was to investigate the association between FIB-4 index and hematoma expansion in patients with intracerebral hemorrhage (ICH). **Methods:** A retrospective review was made of the records of 98 consecutive patients with ICH, separated into two groups according to the FIB-4 index: Group 1 (FIB-4 ≤ 2.67) and Group 2 (FIB-4 > 2.67). The demographic data, admission National Institutes of Health Stroke Scale (NIHSS) scores, hematoma volume on admission and follow-up cranial computed tomography (CT) within 72 h of admission, hematoma extension, mortality, and modified Rankin Scale (mRS) scores at discharge and the first follow-up visit were recorded. **Results:** Group 1 (FIB-4 ≤ 2.67) included 75 patients (28 (37.3%) females, 47 (62.7%) males) and Group 2 (FIB-4 > 2.67) included 23 patients (6 (26.1%) females, 17 (73.9%) males). The results of multivariable regression analysis to evaluate predictors of hematoma expansion showed an independent association of age and FIB-4 index > 2.67 with hematoma expansion. Increasing age was associated with a lower likelihood of hematoma expansion (OR 0.941, 95% CI 0.901–0.983, *p* = 0.012). A FIB-4 index > 2.67 indicated a markedly increased predisposition to hematoma expansion compared to a FIB-4 index ≤ 2.67 (OR 4.12, 95% CI 1.215–13.980, *p* = 0.032). **Conclusions:** The results of this study showed that an elevated FIB-4 index was associated with hematoma expansion. Large-scale prospective studies are needed to confirm this relationship and provide valuable insights for clinical practice.

## 1. Introduction

Hematoma expansion is a frequent and clinically important complication, occurring in approximately one-third of patients with spontaneous intracerebral hemorrhage (ICH), and is a major determinant of subsequent disability and mortality [1]. The identification of factors associated with hematoma expansion is important to be able to guide treatments that will prevent hematoma expansion and improve patient outcomes.

Liver cirrhosis has been recognized as a condition that increases the risk of hemorrhagic stroke, and subsequent hematoma expansion [2]. The underlying mechanisms are thought to involve changes in serum osmolality, increased thrombin production, and systemic inflammation [3,4,5]. However, the effect of occult liver dysfunction on intracerebral hematoma expansion is also of interest [6,7]. Liver fibrosis is a subclinical precursor to advanced chronic liver disease, which often remains asymptomatic [8]. Although the relationship of liver fibrosis with ischemic stroke and other cardiovascular events is well-established, there are very few studies that have specifically investigated its effect on hematoma expansion [6,9].

The Fibrosis-4 (FIB-4) index is a validated and non-invasive biomarker used to estimate subclinical liver fibrosis [10]. Therefore, the aim of this study was to evaluate the association between the FIB-4 index and hematoma expansion in patients with spontaneous ICH.

## 2. Materials and Methods

### 2.1. Study Population

A retrospective review was made of all records in the hospital information management system for patients admitted to the stroke units of two university hospitals with a diagnosis of spontaneous ICH between September 2017 and June 2025. Patients were excluded from the study if they had intracranial hemorrhage secondary to intravenous thrombolytic therapy, hemorrhagic diathesis, sinus vein thrombosis, ischemic stroke, or cerebrovascular malformation. Additionally, those who started reversal treatment were also excluded from the study. In accordance with the AHA/ASA guidelines for spontaneous intracerebral hemorrhage, we aimed to lower systolic blood pressure (SBP) to approximately 140 mmHg (target range 130–150 mmHg) in patients presenting with an SBP between 150 and 220 mmHg, while in patients presenting with SBP > 220 mmHg, blood pressure was reduced using continuous intravenous antihypertensive therapy with frequent blood pressure monitoring [11,12].

### 2.2. Clinical and Functional Assessment

Baseline demographic data, vascular risk factors, antiplatelet or anticoagulant use, and blood pressure value on admission were recorded. Neurological severity on admission was assessed using the National Institutes of Health Stroke Scale (NIHSS). Functional outcomes were evaluated with the modified Rankin Scale (mRS) at discharge and during the first outpatient follow-up. Patients with an mRS score ≥ 3 at follow-up were classified as having poor functional outcomes [13].

### 2.3. Radiological Evaluation

**Figure 1:**
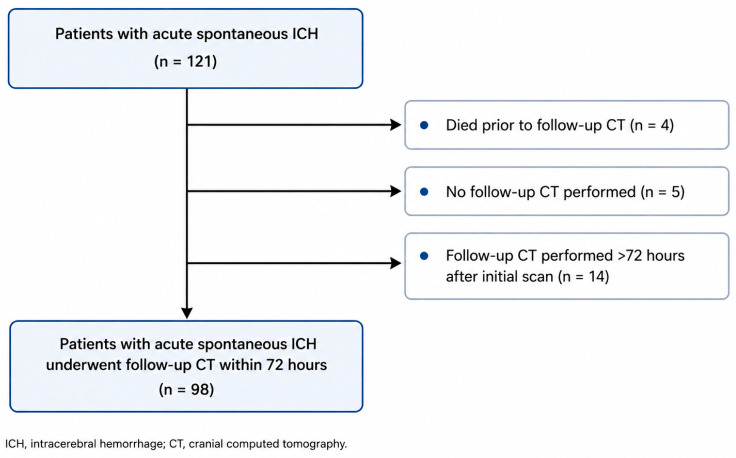
Patient selection flow diagram.

A cranial computed tomography (CT) scan was performed initially and within 72 of admission (mean ± SD: 28.32 ± 22.59 h; range: 6–72 h). Hematoma volumes were calculated using the baseline and follow-up scans obtained within this period. A total of 23 patients were excluded from the analysis: 4 patients died before a follow-up CT could be obtained, 5 patients did not undergo follow-up CT, and 14 patients underwent follow-up CT more than 72 h after the initial scan (Figure 1).

The presence of intraventricular hemorrhage, and CT markers associated with hematoma expansion including blend sign, black hole, island sign, and swirl sign on CT were documented. The presence of a spot sign on CT angiography was also recorded. If gradient-echo sequences or susceptibility-weighted imaging (SWI) were available from previous or current cranial MRI scans, patients were assessed for cerebral amyloid angiopathy (CAA) according to the Boston Criteria 2.0 [14].

In all patients, the hematoma volume was calculated using the ABC/2 method on admission and follow-up cranial CTs performed within 72 h of admission. In this method, the longest diameter (A) and the perpendicular diameter (B) on the slice showing the largest hemorrhage area are measured. The approximate number of slices containing hemorrhage (C) is determined by comparing each CT slice with the largest hemorrhage area: >75% involvement is scored as 1, 25–75% as 0.5, and <25% as 0. The volume (cm^3^) is then calculated as (A × B × C)/2 [15].

Hematoma expansion was defined as an increase in hematoma volume of more than 33% or more than 6 mL on a follow-up brain CT scan performed within 72 h of admission [16].

All radiological measurements were evaluated by the same and blinded researcher.

### 2.4. Assessment of Liver Fibrosis

The FIB-4 index was used to assess liver fibrosis. It was calculated using the following established formula which was derived from peripheral blood parameters on admission [9]: Age (years) × AST (U/L)/platelet (10^9^/L) × √ALT (U/L) [8]. The study participants were separated into two groups according to age-adjusted FIB-4 scores. Patients with FIB-4 values ≤ 2.67 were assigned to Group 1, and those with values > 2.67 were assigned to Group 2 [17]. The cut-off value of 2.67 was determined based on evidence from previous validation studies supporting its ability to identify advanced liver fibrosis, including data derived from Turkish cohorts [18]. There were 6 patients with known liver disease. Three patients in Group 1 were diagnosed with mild hepatosteatosis despite normal liver function tests. Within Group 2, specific pathologies were identified in three patients: hydatid cyst, hepatic adenoma, and hepatosteatosis.

### 2.5. Statistical Analysis

Statistical analyses were performed using SPSS Statistics version 16.0 software (SPSS, Inc., Chicago, IL, USA). Categorical variables are summarized as count (*n*) and percentage (%), and continuous variables as mean ± standard deviation (SD) or median (minimum-maximum) values, depending on distribution. The Chi-square test was applied to compare categorical variables across groups. Continuous variables were analyzed using the independent Student’s *t*-test or one-way analysis of variance (ANOVA) for normally distributed data, and the Mann–Whitney U or Kruskal–Wallis tests were applied to non-normally distributed data. Correlation analyses were performed using Pearson’s or Spearman’s correlation coefficients according to data distribution. Binary logistic regression analysis was performed to identify independent risk factors associated with the dichotomous outcome variable. All analyses were conducted at the 95% confidence level, and statistical significance was defined as *p* < 0.05.

Multicollinearity between independent covariates, particularly between age and the FIB-4 index, was formally evaluated using Tolerance and the Variance Inflation Factor (VIF). A VIF < 2.5 was considered indicative of the absence of significant multicollinearity. The analysis revealed a VIF of [1.249] for age and [1.249] for FIB-4 > 2.67, confirming model stability.

## 3. Results

### Baseline Characteristics

The 98 patients comprised 75 (76.5%) in Group 1 (FIB-4 ≤ 2.67) (28 females [37.3%] and 47 males [62.7%]; mean age 63.4 ± 12.3 years), and 23 (23.5%) in Group 2 (FIB-4 > 2.67) (6 females [26.1%], 17 males [73.9%]; mean age 77 ± 10.2 years). The patients in Group 2 were significantly older than those in Group 1 (*p* ≤ 0.001). The demographic and clinical characteristics of both groups are summarized in Table 1.

No differences between the groups were observed in terms of risk factors, including diabetes mellitus, hypertension, atrial fibrillation, hyperlipidemia, congestive heart failure, coronary artery disease, hyperlipidemia, previous transient ischemic attack or stroke, smoking, alcohol use, history of liver disease, and use of antiplatelet and anticoagulant medication (*p* > 0.05). Systolic blood pressure and NIHSS scores on admission were comparable between the groups (*p* = 0.995 and *p* = 0.286, respectively).

In the records, 43 of the patients participating in the study had CT angiography available. Spot sign was detected in 3 of these CT angiographies.

Patients in the low-FIB-4 group (≤2.67) exhibited a higher prevalence of deep ICH compared to the high-FIB group (57.3% vs. 39.1%), whereas lobar hemorrhage was significantly more frequent in Group 2 (FIB-4 > 2.67) (56.5% vs. 29.3%, *p* = 0.049) (Table 2). Regarding etiology, hypertensive ICH was predominantly observed in the low-FIB-4 group (88.0% vs. 56.5%), whereas cerebral amyloid angiopathy-related ICH was significantly more prevalent among patients with FIB-4 index > 2.67 (34.8% vs. 10.7%, *p* = 0.03, Table 2).

Hematoma expansion was defined as an increase in hematoma volume of more than 33% or more than 6 mL on a follow-up brain CT scan performed within 72 h of admission [16]. In 4 of the 35 patients, there was a 6 mL increase in the hematoma; in 28 patients, there was a 33% increase; and in 3 patients, both were present.

No significant difference was determined between the groups in respect of baseline and within 72 h follow-up hematoma volumes. The frequencies of intraventricular hemorrhage, irregular hematoma shape, and specific non-contrast CT expansion markers (blend, island, satellite, and black hole signs) were similar in both groups (all *p* > 0.05). Although hematoma expansion occurred more frequently in patients with FIB-4 > 2.67 compared to those with FIB-4 ≤ 2.67 (52.2% vs. 30.7%), the difference was not statistically significant (*p* = 0.060).

Clinical outcomes, including in-hospital mortality, mRS scores at discharge, and poor functional status at follow-up were comparable between Group 1 and Group 2 (Table 1).

The results of multivariable regression analysis evaluating risk factors for hematoma expansion showed that age and FIB-4 index > 2.67 were independently associated with hematoma expansion. Older age was associated with a low likelihood of hematoma expansion (OR 0.941, 95% CI 0.901–0.983, *p* = 0.012). Patients with a FIB-4 index > 2.67 had significantly increased odds of hematoma expansion compared to those with a FIB-4 index ≤ 2.67 (OR 4.12, 95% CI 1.215–13.980, *p* = 0.032, Table 3).

## 4. Discussion

In this retrospective cohort study, the FIB-4 index was used as a non-invasive surrogate marker of liver fibrosis to investigate the relationship between subclinical liver disease and hematoma expansion. The prevalence of lobar hemorrhage and cerebral amyloid angiopathy was more common in the FIB-4 > 2.67 group. The results of the multivariable regression analysis revealed two significant independent risk factors: while older age was paradoxically associated with a lower likelihood of hematoma expansion, an elevated FIB-4 index was strongly linked to an increased risk of this outcome.

The impact of overt liver disease and elevated hepatic enzymes on hematoma formation and expansion has been well-documented [19,20,21,22]. However, this relationship remains less clear in the context of subclinical liver disease. While the current study findings are consistent with some previous research suggesting an association between liver fibrosis and hematoma expansion [4,16], there are also studies that have failed to demonstrate such a link [5]. The underlying pathophysiology of liver fibrosis, similar to that of advanced hepatic disease, may involve a complex interplay of coagulopathy, vascular inflammation, and endothelial dysfunction [20,21,22]. Whether these mechanisms fully account for the observed risk in subclinical stages remains a hypothesis that warrants further prospective validation.

Regarding clinical outcomes, the in-hospital mortality rates of this study were comparable between the groups, and liver fibrosis indices were not independently associated with mortality. This partially contrasts with the findings of Parikh et al., who reported a significant association between fibrosis indices and the 90-day mortality rate [6]. Although Tan et al. reported that subclinical derangements in individual enzymes such as AST and ALP were correlated with poor outcomes after ICH in univariate models, these associations were seen to generally disappear after adjusting for confounders [23].

Advanced age is a well-established predictor of poor clinical outcomes after ICH, although its relationship with hematoma expansion remains inconsistent. In the current study, increasing age was paradoxically associated with a lower likelihood of hematoma expansion, despite the high-FIB-4 cohort being significantly older. Previous large-scale observational studies have reported that advanced age was more strongly linked to overall mortality and poor functional recovery rather than to active hematoma expansion itself [24,25,26]. The higher propensity for hematoma expansion observed in relatively younger patients may reflect more active bleeding dynamics and pronounced baseline blood pressure variability. Furthermore, this disparity most likely mirrors shifting ICH etiologies across age groups. Age-related alterations in vascular compliance and hemostatic responses may also modulate this bleeding behavior. Ultimately, the current study results suggest that age is not an isolated predictor of HE, but that it interacts dynamically with underlying vascular pathologies and systemic markers such as FIB-4, underscoring the necessity for multidimensional risk stratification in ICH.

Another notable finding was the significantly higher prevalence of lobar hemorrhage and cerebral amyloid angiopathy (CAA) among patients with high FIB-4 index, supporting a potential link between systemic fibrotic burden and cerebral small vessel pathology. A previous study has indicated that the FIB-4 index correlates positively with larger hematoma volumes and worse clinical outcomes in patients with lobar ICH but not with deep ICH [7]. Furthermore, lobar hemorrhages have been associated with greater expansion rates and larger volumes [27]. These findings suggest that FIB-4 may serve not only as a marker of hepatic fibrosis but also as an indicator of vascular vulnerability within the cerebral microcirculation [5,6,7]. The observed relationship with both lobar hemorrhage and cerebral amyloid angiopathy further strengthens the hypothesis that shared pathophysiological mechanisms, such as endothelial dysfunction and chronic inflammation, may underlie this association [7,27,28,29]. Consequently, FIB-4 may have a potential benefit in risk stratification and prognostic assessment in patients with lobar ICH [6,7,23,27].

This study had several limitations. One was the retrospective design and relatively small sample size, particularly in the high-FIB-4 group (*n* = 23). This limited the number of total hematoma expansion events (*n* = 35), introducing a risk of statistical overfitting when controlling for numerous covariates. To mitigate this, we utilized a parsimonious regression model focusing on key clinical drivers. Although key imaging metrics like the CT spot sign or exact time-to-CT window could not be integrated into the final multivariable regression due to sample size constraints, univariate checks showed no significant baseline imbalances for non-contrast CT markers between the groups. Larger multicenter prospective cohorts are strictly necessary to validate these findings using high-dimensional covariate adjustments. There may be measurement errors during the ABC/2 method. This measurement method may not be randomly distributed across groups.

## 5. Conclusions

In conclusion, an elevated FIB-4 index (>2.67) upon admission was independently associated with a higher risk of hematoma expansion in patients with spontaneous ICH. Routine calculation of the FIB-4 index in the acute setting may provide preliminary prognostic insights, potentially aiding clinicians in identifying patients who require closer monitoring. However, whether these findings can safely guide specific therapeutic strategies, such as intensive blood pressure management or hemostatic interventions, remains to be determined. Future large-scale, prospective multicenter studies are warranted to validate these findings and elucidate the underlying mechanisms linking hepatic biomarkers with cerebral microvascular outcomes.

## Figures and Tables

**Table 1:** Demographic and clinical characteristics of the patients.

	FIB-4 ≤ 2.67 *n* = 75 (76.5)	FIB-4 > 2.67 *n* = 23 (23.5)	*p*
**Age, (years)** mean ± SD	63.4 ± 12.3	77.0 ± 10.2	<0.001 *
**Sex, *F*/*M* (%)**	28 (37.3)/47 (62.7)	6 (26.1)/17 (73.9)	0.322
**Risk Factors**			
Hypertension, *n* (%)	53 (70.7)	13 (56.5)	0.206
Diabetes mellitus, *n* (%)	20 (26.7)	4 (17.4)	0.421
Atrial fibrillation, *n* (%)	3 (4.0)	2 (8.7)	0.334
Dyslipidemia, *n* (%)	10 (13.3)	1 (4.3)	0.450
Coronary artery disease, *n* (%)	11 (14.7)	3 (13.0)	0.999
Congestive heart failure, *n* (%)	8 (10.7)	1 (4.3)	0.681
History of stroke, *n* (%)	10 (13.3)	6 (26,1)	0.148
Smoking, *n* (%)	16 (21.3)	2 (8.7)	0.170
Alcohol, *n* (%)	3 (4.0)	2 (8.7)	0.304
Liver disease, *n* (%)	3 (4.0)	3 (13.0)	0.139
**Drug Usage**			
**Antiplatelet drugs, *n* (%)**	23 (30.7)	5 (21.7)	0.407
ASA, *n* (%)	13 (17.1)	3 (13.6)	
Clopidogrel, *n* (%)	5 (6.6)	2 (9.1)	
ASA + Clopidogrel, *n* (%)	5 (6.6)	0 (0)	
**Anticoagulant drugs, *n* (%)**	4 (5.3)	4(17.4)	0.085
Warfarin, *n* (%)	2 (2.6)	2 (9.1)	
DOAC, *n* (%)	1 (1.3)	2 (9.1)	
LMWH, *n* (%)	1 (1.3)	0 (0)	
Admission SBP, mean ± SD	176.27 ± 32.90	165.45 ± 31.53	0.995
Initial SBP > 140, *n* (%)	53 (70.7)	13 (56.5)	0.206
Initial SBP > 180, *n* (%)	41 (54.7)	11 (47.8)	0.565
Admission NIHSS, mean ± SD	8.12 ± 5.57	9.04 ± 4.74	0.286
Mortality at hospital, *n* (%)	12 (16.0)	4 (17.4)	0.875
mRS at discharge, mean ± SD	3.13 ± 1.73	3.74 ± 1.45	0.296
mRS > 2 at follow up, *n* (%)	36 (47.4)	12 (57.1)	0.428
mRS at follow up, mean ± SD	2.92 ± 1.81	3.36 ± 1.87	0.496

* *p* < 0.05. Abbreviations: SD, standard deviation; SBP, systolic blood pressure; ASA, acetylsalicylic acid; DOAC, dual oral anticoagulant; LMWH, low-molecular-weight heparin.

**Table 2:** Radiological findings of the patients.

	FIB-4 ≤ 2.67 *n* = 75 (76.5)	FIB-4 > 2.67 *n* = 23 (23.5)	*p*
**Location of ICH**			
Deep, *n* (%)	43 (57.3)	9 (39.1)	0.049
Lobar, *n* (%)	22 (29.3)	13 (56.5)	
Infratentorial, *n* (%)	10 (13.3)	1 (4.3)	
**ICH etiology**			
Hypertensive, *n* (%)	66 (88.0)	13 (56.5)	0.003
CAA, *n* (%)	8 (10.7)	8 (34.8)	
Mix type, *n* (%)	1 (1.3)	2 (8.7)	
Time from ICH symptom onset to first cranial CT -Median (IQR)	4 (1–11.5)	6 (1.5–15.5)	0.47
Admission hematoma volume -Median (IQR)	3.6 (1.8–9.62)	7.2 (2.05–10.22)	0.315
Hematoma volume within 72 h -Median (IQR)	2.95 (1.5–9.3)	7.0 (1.20–10.20)	0.310
Hematoma expansion, *n* (%)	23 (30.7)	12 (52.2)	0.060 *
Intraventricular hemorrhage, *n* (%)	26 (34.7)	9 (39.1)	0.696
Irregular-shaped hematoma, *n* (%)	24 (32.0)	7 (30.4)	0.888
NCCT predictors			
Blend sign, *n* (%)	11 (14.7)	4 (17.4)	0.747
Island sign, *n* (%)	14 (18.4)	1 (4.5)	0.181
Satellite sign, *n* (%)	7 (9.3)	3 (13.0)	0.695
Black hole sign, *n* (%)	5 (6.7)	0 (0)	0.588
In-hospital mortality, *n* (%)	12 (16.0)	4 (17.4)	0.875
mRS at discharge, mean ± SD	3.13 ± 1.73	3.74 ± 1.45	0.296
mRS > 2 at follow up, *n* (%)	36 (47.4)	12 (57.1)	0.428
mRS at follow up, mean ± SD	2.92 ± 1.81	3.36 ± 1.87	0.496

* *p* < 0.05. Abbreviations: ICH: Intracerebral hemorrhage, CAA: cerebral amyloid angiopathy, SD: Standard deviation, mRS: modified Rankin Scale, NCCT: non-contrast computed tomography.

**Table 3:** Multiple regression analysis of the groups for hematoma expansion.

	OR Exp(B)	95% CI	*p*
Age	0.941	0.901–0.983	0.012 *
FIB-4 > 2.67	4.120	1.215–13.980	0.032 *
Admission NIHSS	1.075	0.980–1.180	0.112

* *p* < 0.05. Abbreviations: OR, Odds Ratio; 95% CI, 95% Confidence Interval; NIHSS, National Institutes of Health Stroke Scale.

## Data Availability

The datasets analyzed in the study are available from the corresponding author upon reasonable request.
